# Networks and caregiving: understanding resilience, wellbeing, and burden among dementia care partner networks

**DOI:** 10.1093/geroni/igaf122.569

**Published:** 2025-12-31

**Authors:** M Aaron Guest, Allie Peckham, Sage Sadow, Keenan Pituch, Casey Davies, Giselle Reyes

**Affiliations:** Arizona State University, Phoenix, Arizona, United States; Arizona State University, Phoenix, Arizona, United States; Arizona State University, Phoenix, Arizona, United States; Arizona State University, Phoenix, Arizona, United States; Arizona State University, Phoenix, Arizona, United States; Arizona State University, Phoenix, Arizona, United States

## Abstract

Research on dementia caregiving often focuses on the care partner and care recipient dyad, overlooking the broader social networks that shape caregiver well-being, resilience, and burden. This study addresses this gap by examining how social network composition influences caregiver outcomes. Using an egocentric network approach, we conducted guided interviews with 107 unpaid caregivers of individuals living with dementia in Arizona and Nevada. Data collected included caregiver and care recipient demographics, social network satisfaction, caregiver stress resilience, well-being, and social network characteristics, including demographic similarity and perceptual affinity. Caregivers were predominantly female (87%) and white (80.1%), with networks averaging 9.6 members (SD = 3.97). Participants reported high perceptual affinity with their networks (.71) but lower demographic similarity (.52) and strong tie strength (.78). Multiple regression analyses revealed that perceptual affinity was positively associated with resilience (β = .33, SE = .11) and well-being (β = .20, SE = .10) and negatively associated with burden (β = -.39, SE = .10). Additionally, total network members (β = .19, SE = .07) and caregiver tenure (β = .19, SE = .09) were positively associated with resilience. No other associations were significant. Findings highlight the importance of broader social networks in caregiving, demonstrating that perceptual affinity—shared commonalities and interest within networks—plays a key role in enhancing resilience and well-being while reducing burden. These insights underscore the need for interventions that strengthen meaningful social connections to support caregivers more effectively and additional research to identify the exact types of perceptual affinity that improve outcomes.

